# Ratios of Four *STAT3* Splice Variants in Human Eosinophils and Diffuse Large B Cell Lymphoma Cells

**DOI:** 10.1371/journal.pone.0127243

**Published:** 2015-05-18

**Authors:** Keren B. Turton, Douglas S. Annis, Lixin Rui, Stephane Esnault, Deane F. Mosher

**Affiliations:** 1 Department of Biomolecular Chemistry, University of Wisconsin-Madison, Madison, WI, United States of America; 2 Department of Medicine at University of Wisconsin-Madison, Madison, WI, United States of America; Cancer Research Centre of Lyon, FRANCE

## Abstract

Signal transducer and activator of transcription 3 (STAT3) is a key mediator of leukocyte differentiation and proliferation. The 3' end of *STAT3* transcripts is subject to two alternative splicing events. One results in either full-length STAT3α or in STAT3β, which lacks part of the C-terminal transactivation domain. The other is at a tandem donor (5') splice site and results in the codon for Ser-701 being included (S) or excluded (ΔS). Despite the proximity of Ser-701 to the site of activating phosphorylation at Tyr-705, ΔS/S splicing has barely been studied. Sequencing of cDNA from purified eosinophils revealed the presence of four transcripts (S-α, ΔS-α, S-β, and ΔS-β) rather than the three reported in publically available databases from which ΔS-β is missing. To gain insight into regulation of the two alternative splicing events, we developed a quantitative(q) PCR protocol to compare transcript ratios in eosinophils in which STAT3 is upregulated by cytokines, activated B cell diffuse large B cell Lymphoma (DLBCL) cells in which STAT3 is dysregulated, and in germinal center B cell-like DLBCL cells in which it is not. With the exception of one line of activated B cell DLCBL cells, the four variants were found in roughly the same ratios despite differences in total levels of *STAT3* transcripts. S-α was the most abundant, followed by S-β. ΔS-α and ΔS-β together comprised 15.6±4.0 % (mean±SD, n=21) of the total. The percentage of *STAT3β* variants that were ΔS was 1.5-fold greater than of *STAT3α* variants that were ΔS. Inspection of Illumina’s “BodyMap” RNA-Seq database revealed that the ΔS variant accounts for 10-26 % of *STAT3* transcripts across 16 human tissues, with less variation than three other genes with the identical tandem donor splice site sequence. Thus, it seems likely that all cells contain the S-α, ΔS-α, S-β, and ΔS-β variants of STAT3.

## Introduction

Signal transducer and activator of transcription 3 (STAT3) is a transcription factor in the Janus kinase (JAK)/STAT signaling pathway. Its aberrant expression is a contributing factor in some cancers (for a review see [1]). Ablation of the gene is embryonically lethal in mice, and tissue-specific knockout of *STAT3* leads to a number of informative phenotypes [2,3], including increased sensitivity to endotoxic shock. Activity of STAT3 is modulated by phosphorylation events near the C-terminus of the protein, the region that controls protein dimerization and lifetime after activation [4]. Tyr-705 is phosphorylated by several kinases, including JAK1/2 [5], and Ser-714 and -727 are phosphorylated by GSK3 [6]. Dimerized STAT3 associates with GAS elements in DNA to initiate transcription of immediate early genes [7].

**Fig 1:**
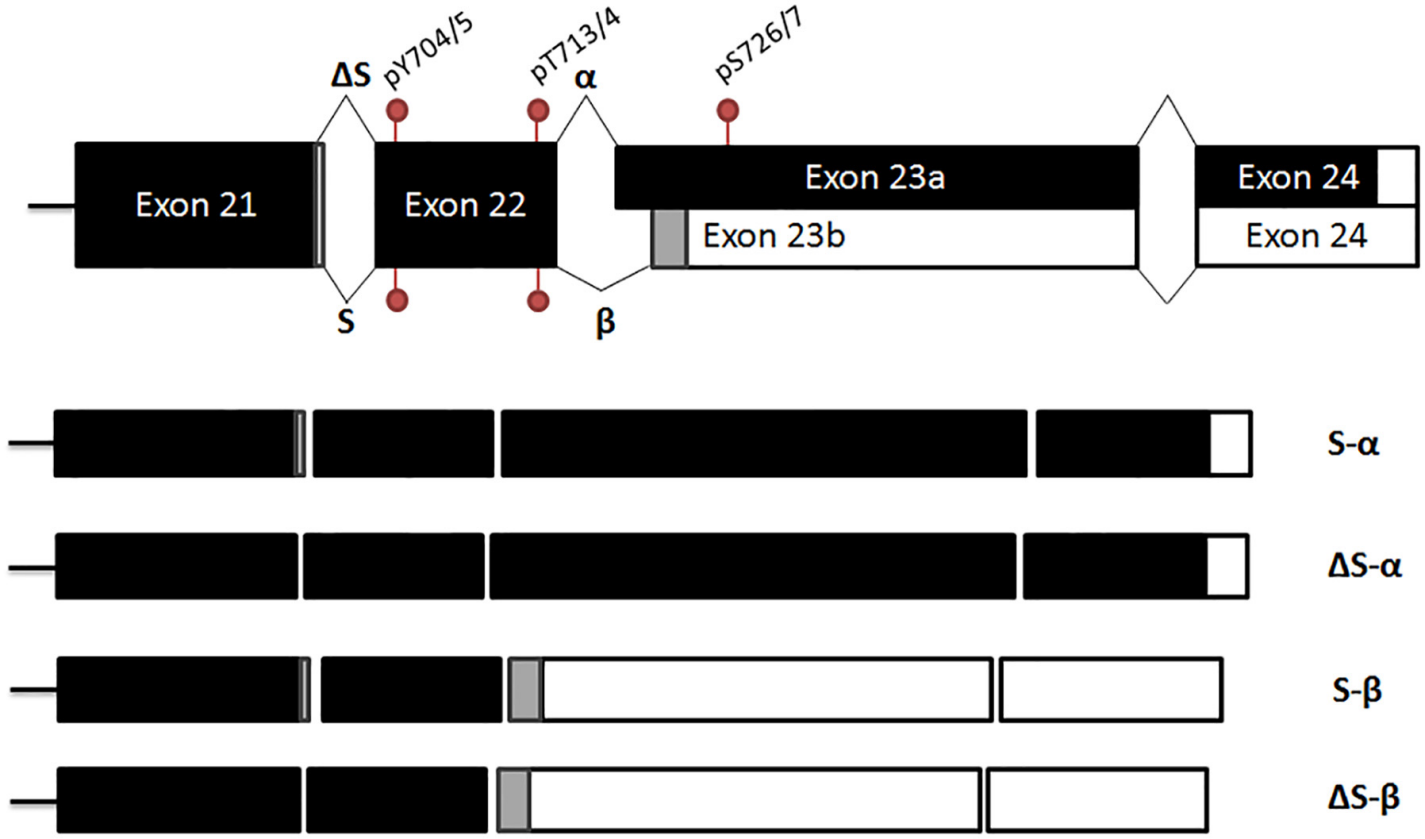
Schematic of the four *STAT3* splice variants. The splice sites are near the 3' end of the transcript, so only the exons closest to this end are shown for simplicity. The non-coding 3’ UTR is shown in white, with grey representing different coding sequences as a result of alternative splicing. Phosphorylation sites present in the translated form are also shown on the transcript.

Two splice sites near the 3ˈ end of the *STAT3* gene determine the coding sequence, resulting in four possible splice variants (Fig 1). The *STAT3* α/β splice site is well-characterized [3], with an out-of-frame alternate acceptor site in exon 23 leading to either inclusion of a 55-residue transactivation domain (α) or 7 unique residues (β). As with alternative splicing of many genes [8], α/β alternative splicing is of great functional importance. Lack of STAT3α leads to premature death in mice [9]. STAT3β modulates inflammation [10] and regulates expression of genes controlling protein metabolism and transport in HEK-293 cells differently compared to STAT3α [11]. Using morpholinos to redirect splicing, Zammarchi *et al*. [12] showed that STAT3α is oncogenic, while STAT3β has tumor suppressive functions.

Much less is known about the significance of a short-range (tandem) donor (5') splice site, here called ΔS/S because the absence of three nucleotides leads to Ser-701 being excluded from the protein. *STAT3* mRNA with and without these three nucleotides was first described in 1995 for *STAT3β* [13] and subsequently shown to arise from alternative splicing at the donor site [14]. Not only are these the only papers on ΔS/S splicing but databases are hit-or-miss. For instance, despite the fact that ΔS was first described in *STAT3β* transcripts [13], RefSeq [15] describes validated sequences for only S-α, S-β, and ΔS-α (NM_003150.3), and UniProt describes the S-α (770 residues), ΔS-α (769 residue) and S-β (722 residues), but not the ΔS-β (721 residues) variant of human STAT3.

Eosinophils are one of the cell types in which STAT3β was first described [9]. When we sequenced eosinophil cDNA with primers specific for α and β variants, we found sequences for all four predicted splice variants of *STAT3;* S-α, ΔS-α, S-β, and ΔS-β. Following stimulation of eosinophils with IL5 (interleukin 5)-family cytokines, STAT3 becomes activated as assessed by phosphorylation of Tyr-705 [16]. To examine accompanying changes in *STAT3* mRNAs following cytokine stimulation, we developed primer-specific quantitative(q) PCR assays for measurement of levels and ratios of *STAT3* splice variant transcripts in eosinophils without or with cytokine stimulation. As context for the eosinophil results, the qPCR analyses were extended to cell lines derived from two subtypes of DLBCL (diffuse large B cell lymphoma): activated B cell-like (ABC) DLBCL, which requires activated STAT3 for growth and in which STAT3 is known to be highly-expressed, and germinal center B cell-like (GCB) DLBCL in which STAT3 expression is much lower [17]. Finally, we determined α/β and ΔS/S ratios in 16 human tissues based on RNA-Seq data in Illumina’s “BodyMap” database (GEO accession GSE30611; [18]). We conclude that the two splicing events generate four splice variants of *STAT3* that probably are present at similar ratios in all cells. Further, our data suggest that splicing at the ΔS/S and α/β sites is coordinated. STAT3 is becoming increasingly recognized as having diverse functions in addition to transcriptional activity [19,20], raising the question of how STAT3 splice variants may contribute to such heterogeneous functionality. STAT3α and β are already known to have overlapping but non-redundant functions. A complete understanding of STAT3 activities likely will need to also take into account the ΔS/S site.

## Results

### All four *STAT3* splice variants are present in eosinophils and DLBCL

**Fig 2:**
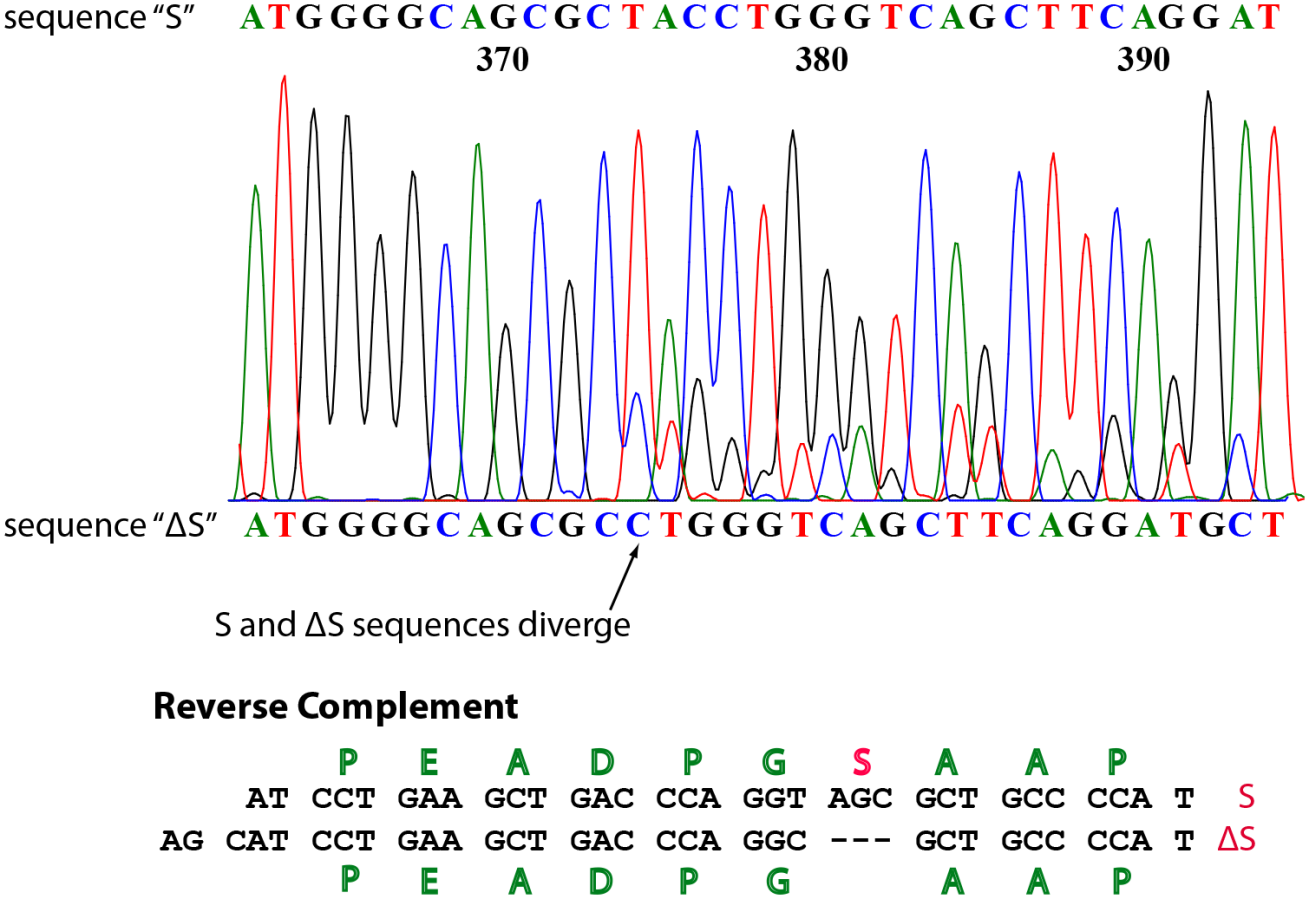
*STAT3* S-β and ΔS-β splice variant transcripts are present in eosinophils. PCR products were cloned into pET-Elmer vectors, amplified in *E*. *coli* and sequenced. The electrophoretogram depicts sequencing data from PCR amplification with a reverse primer specific for *STAT3β*. The amino acids encoded by the complementary sequence are shown below.

Amplification of eosinophil cDNA by PCR with a 5' primer to a sequence in exon 19 encoding the start of the SH2 domain and a 3' primer specific for *STAT3β* yielded products that spanned the ΔS/S site. The sequences of the PCR products diverged at the position of the codon for Ser-701 (Fig 2). The minor sequence was that of the ΔS variant. Similar amplifications with a primer specific for *STAT3α* yielded products that also had a minor sequence corresponding to the ΔS variant (not shown). The four amplicons were cloned and re-sequenced, yielding plasmids encoding the four variants. PCR was also done with common upstream (exon 19) and downstream (exon 23) primers, products were cloned, and plasmids specific for each of the four sequences were identified by sequencing and produced for use as standards for qPCR. We also found sequences for the four possible *STAT3* splice variants when we did similar amplifications of cDNAs of ABC (Oci-LY10) and GC (Oci-LY1) DLBCL cell lines.

### Establishment of qPCR assays for the four variants and total *STAT3* mRNA

**Fig 3:**
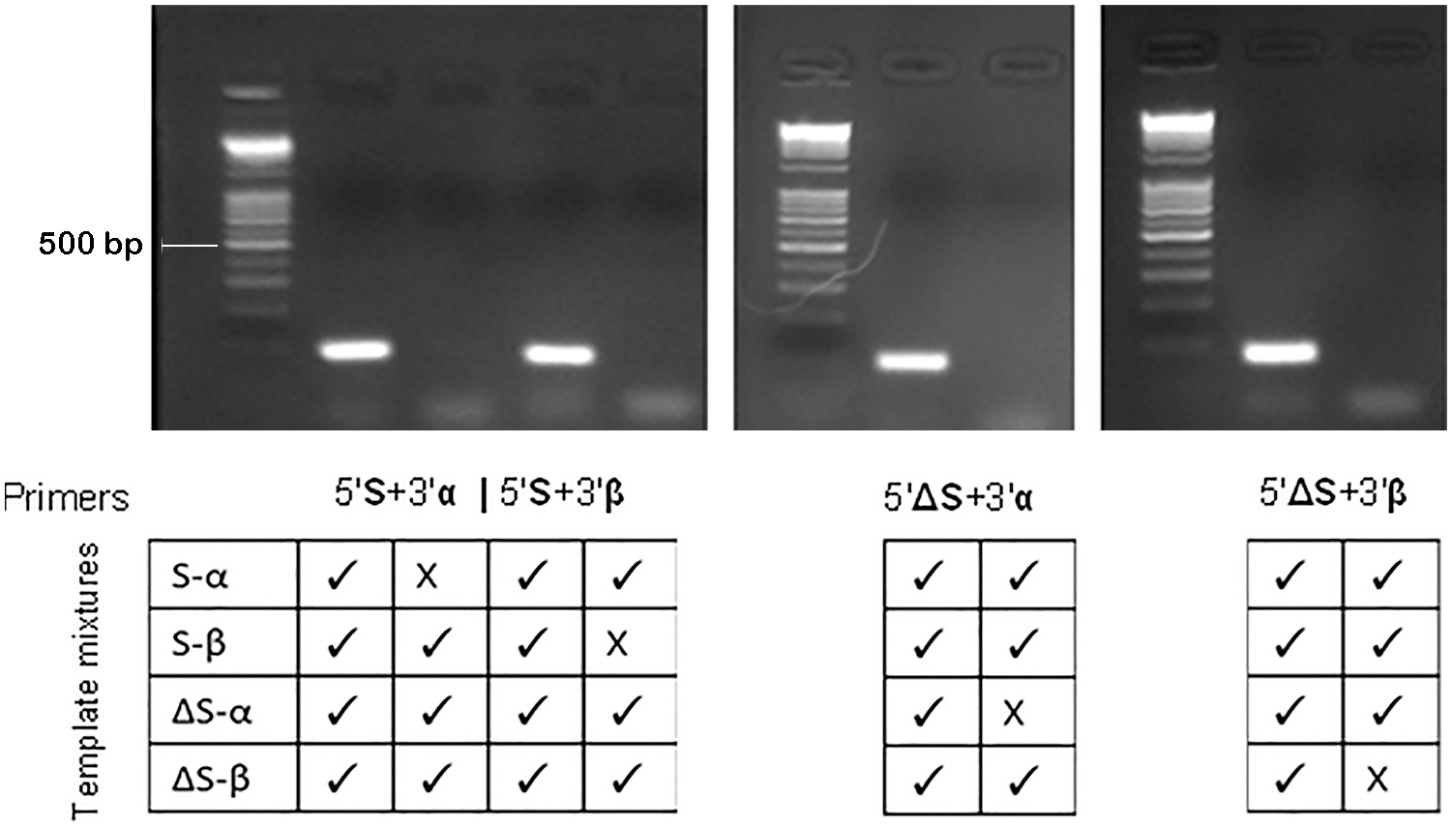
Validation of primer specificity. The specificity of primers uniquely amplifying the four *STAT3* splice variants was validated using mixed isoforms as template in PCR. An annealing temperature of 66°C was required to exclude S-α and S-β amplifications from the ΔS-α and ΔS-β reactions, respectively. DNA ladder is exACTGene 1 Kb Plus DNA Ladder (Fisher Scientific International Inc., Waltham, MA).

**Fig 4:**
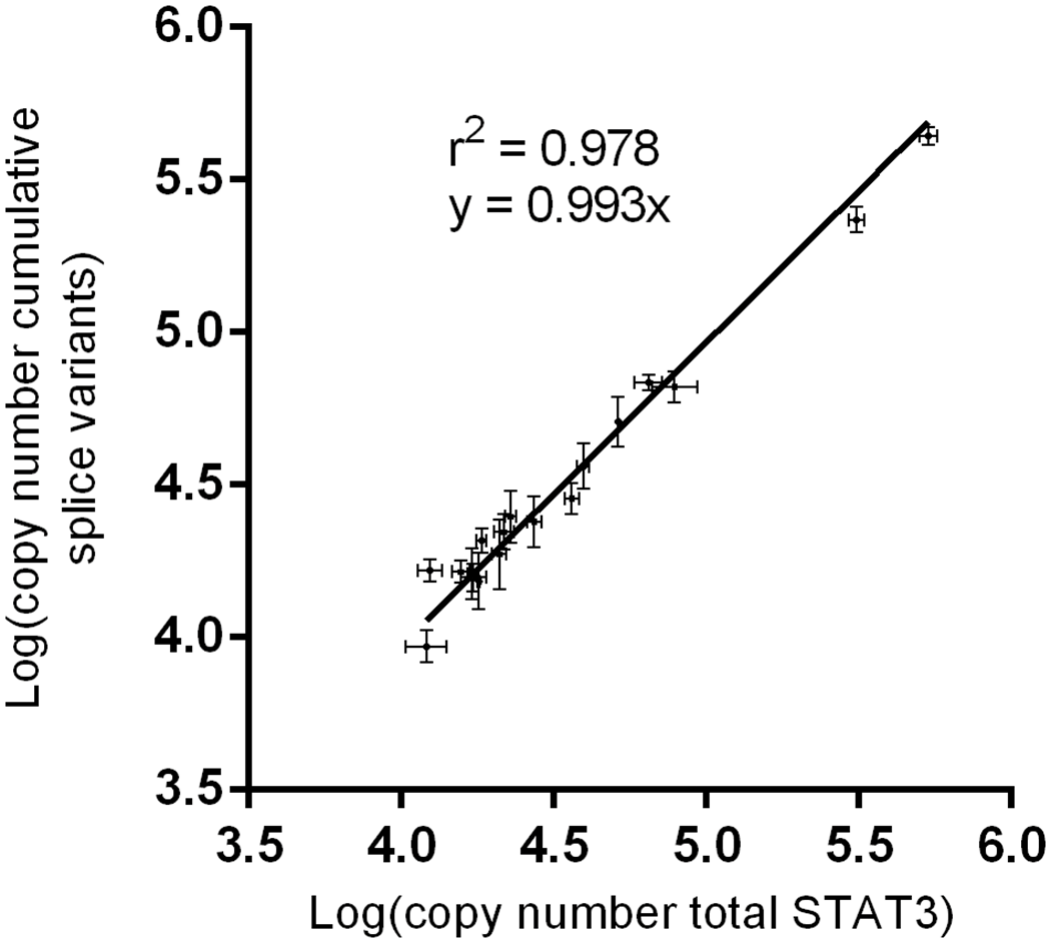
Pan-*STAT3* primer quantification was compared to the summation of the four splice variants. Quantification was performed for 18 eosinophil or DLBCL samples to validate the absolute qPCR protocol. Each point is plotted with its CV and represents the average of three replicates.

Primers designed to be specific for S, ΔS, α, and β were tested to identify combinations and amplification conditions that amplify each splice variant specifically (Table 1). Specific amplification was assessed by PCR using a mix of all four cloned splice variants as template DNA, and a mix of the three splice variants that should *not* be amplified by the specific primer combination as a negative control (Fig 3). An annealing temperature of 66°C, which was required for S and ΔS splice variants to be amplified specifically, was associated with a replication efficiency of ~80%. This low efficiency limits the qPCR assay to absolute quantification (in comparison to a standard) of concentrated cDNA solutions. A more efficient primer pair with the forward primer spanning exons 19 and 20 and the reverse in exon 21 (present in all *STAT3* variants) was designed for both quantification of total *STAT3* relative to the reference gene glucuronidase-β (*GUSB*) and absolute quantification of total *STAT3* mRNA. Annealing was at 60°C, and the replication efficiency was ~100%. In absolute quantification assays, the linear regression value (r^2^) for 18 samples was 0.978 between logs of the sum of the four splice variants determined by variant specific qPCR and total *STAT3* with a slope of 0.99 (Fig 4). A slope of 1 falls within the 90% confidence interval.

**Table 1:** Primers used for quantitative PCR analysis.

Transcript Amplified	Forward primer	Reverse Primer	Amplicon size (bp)
***qPCR primers for absolute quantification***			
***STAT3* S-α**	5ˈ- GAA GCT GAC CCA GG**T AGC** -3ˈ	5ˈ-CAT CGG CAG GTC AAT GGT A-3ˈ	93
***STAT3* S-β**	5ˈ- GAA GCT GAC CCA GG**T AGC** -3ˈ	5ˈ-CAA ACT GCA TCA ATG AAT GGT GTC-3ˈ	77
***STAT3* ΔS-α**	5ˈ- TGA AGC TGA CCC AGG **CG** -3ˈ	5ˈ-CAT CGG CAG GTC AAT GGT A -3ˈ	91
***STAT3* ΔS-β**	5ˈ- TGA AGC TGA CCC AGG **CG** -3ˈ	5ˈ-CAA ACT GCA TCA ATG AAT GGT GTC-3ˈ	75
***qPCR primers for relative quantification***			
**Pan-*STAT3***	5ˈ- GAG AAG GAC ATC AGC GGT AAG -3ˈ	5ˈ- AGT GGA GAC ACC AGG ATA TTG -3ˈ	137
***GUSB***	5ˈ- CTC ATT TGG AAT TTT GCC GAT T -3ˈ	5ˈ- CCG AGT GAA GAT CCC CTT TTT A -3ˈ	81

### Splice variant ratios when *STAT3* transcription is induced in eosinophils

**Fig 5:**
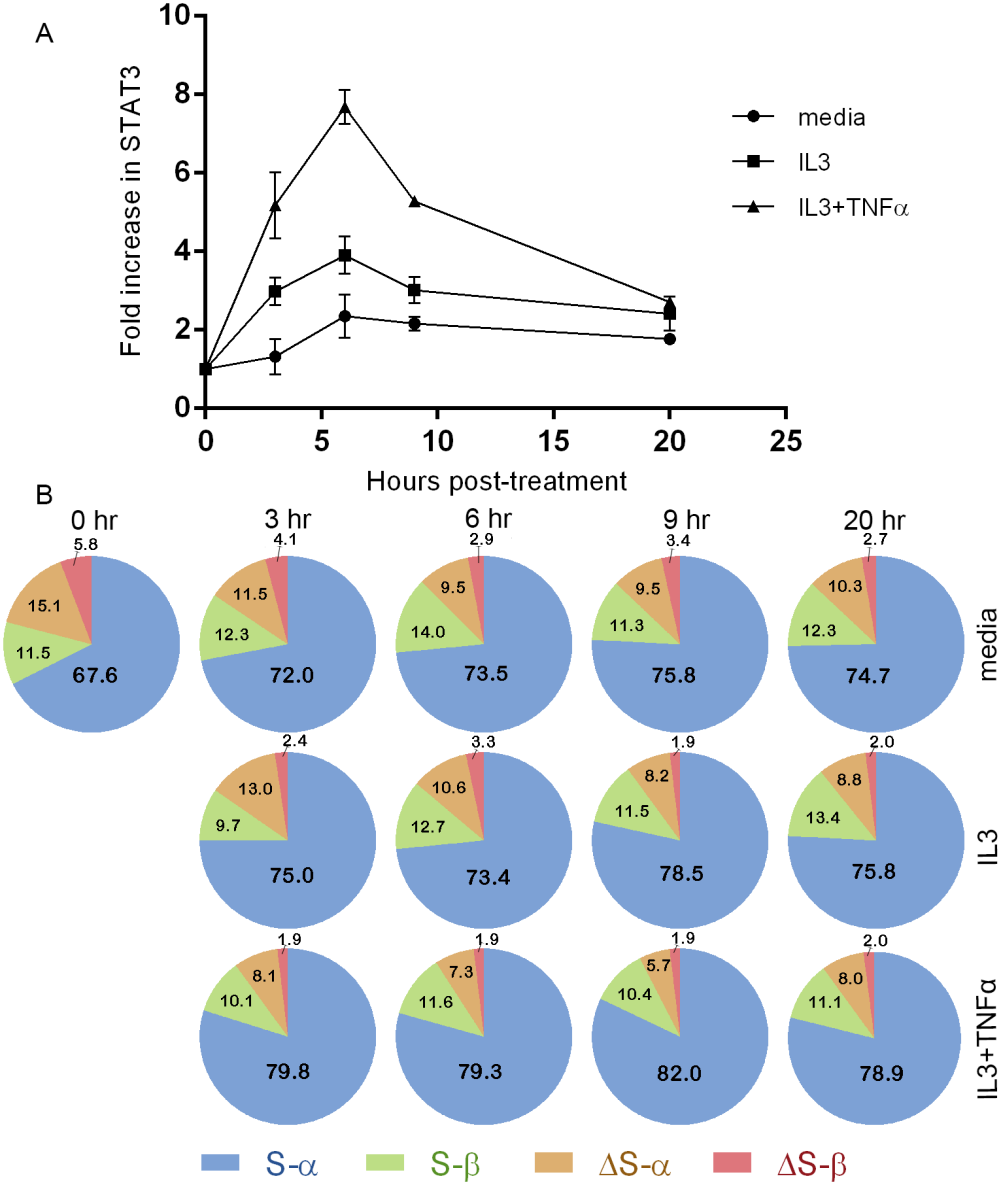
Splice variant ratios of eosinophil *STAT3* change little when *STAT3* transcription is induced. **(A)** Relative quantification qPCR was used to determine fold changes in overall *STAT3* levels in eosinophils over time under different conditions determined using *GUSB* as a reference gene. **(B)** Pie charts of *STAT3* splice variants in untreated and treated eosinophils. Absolute quantification qPCR was used to determine proportions of *STAT3* splice variants. Percentages of each splice variant are shown. Data represent averages determined from at least three reactions, with standard deviations shown for (A). IL3 = interleukin 3. TNFα = tumor necrosis factor α.

Overall changes in *STAT3* transcription were analyzed using relative qPCR (ΔΔCt method [21] with *GUSB* as the reference gene) in eosinophils incubated in suspension without or with interleukin 3 (IL3) and/or tumor necrosis factor α (TNFα), with biological duplicates of the 6 hr time-point (media, IL3, and IL3+TNFα). There was a 7.7-fold increase in *STAT3* mRNA levels in IL3+TNFα-treated eosinophils after 6 hours, a 3.9-fold increase with IL3 alone, and a 2.3-fold increase without cytokine (Fig 5A). Levels in all samples dropped after 6 hours.

The fold increase in *STAT3* relative to GUSB in IL3+TNFα stimulated eosinophils was significantly higher than both that of IL3 and untreated eosinophils at the 6 hr time point (p<0.05, paired t-tests). The fold increases in individual splice variants in IL-3+TNFα-activated eosinophils, calculated based on the ratio of the four variants at the 6-hour time point were: *STAT3* S-α, 9.2-fold; S-β, 6.4-fold; ΔS-α, 3.4-fold and ΔS-β, 3.2-fold. These data indicate that activation with IL-3+TNFα perturbs the ratio of the 4 splicing variants with enrichment in S-α and decrease of the ΔS versus the S variants.

### *STAT3* splice variant compositions of ABC and GBC DLBCL cells

**Fig 6:**
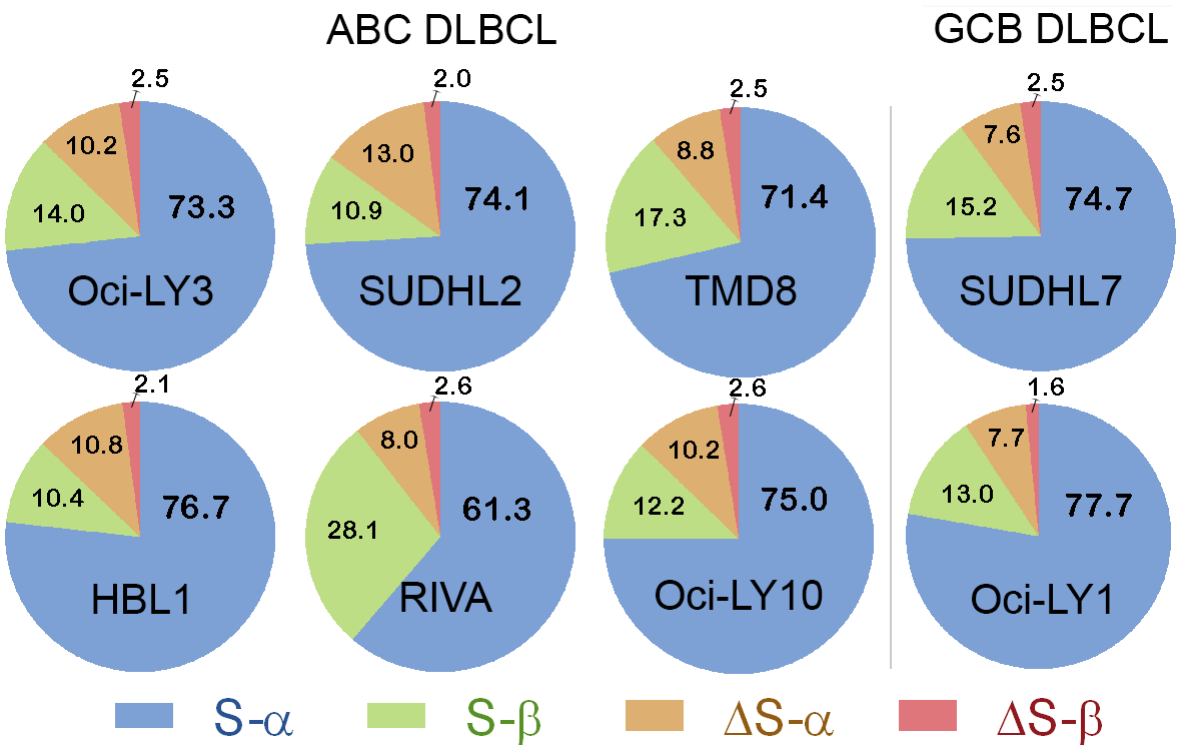
Pie charts of *STAT3* splice variants in DLBCL. Splice variant ratios in DLBCL differ minimally, except for RIVA. Absolute quantification qPCR was used to determine proportions of *STAT3* splice variants in each cell line. Percentages of each splice variant are shown. Six samples are activated B cell DLBCL and two are germinal center B cell-like DLBCL. Data represent averages determined from at least three assays for duplicate reactions.

Absolute quantification qPCR demonstrated little difference in the ratios of *STAT3* splice variants among ABC and GCB DLBCL cells with one exception (Fig 6). RIVA ABC cells had a significantly different (p<0.05) proportion of *STAT3* S-β compared to the other DLBCL cell lines.

### ΔS/S and α/β splicing are interdependent

We observed a bias in *STAT3* splice variant ratios: while α is more abundant than β, and S is more common than ΔS, more β splice variants were ΔS than α splice variants, that is, the percentage of β splice variants lacking the Ser-701 codon across all samples was 17±6% (mean±SD), while the percentage for α was 11±3% (n of 21, p<0.005). Thus, the splice sites are interdependent in that there is intragenic coordination of the ΔS/S and α/β splice sites, a phenomenon described first in *C*. *elegans’ slo-1* gene [22].

### α/β and ΔS/S *STAT3* splice variants in human tissues

**Fig 7:**
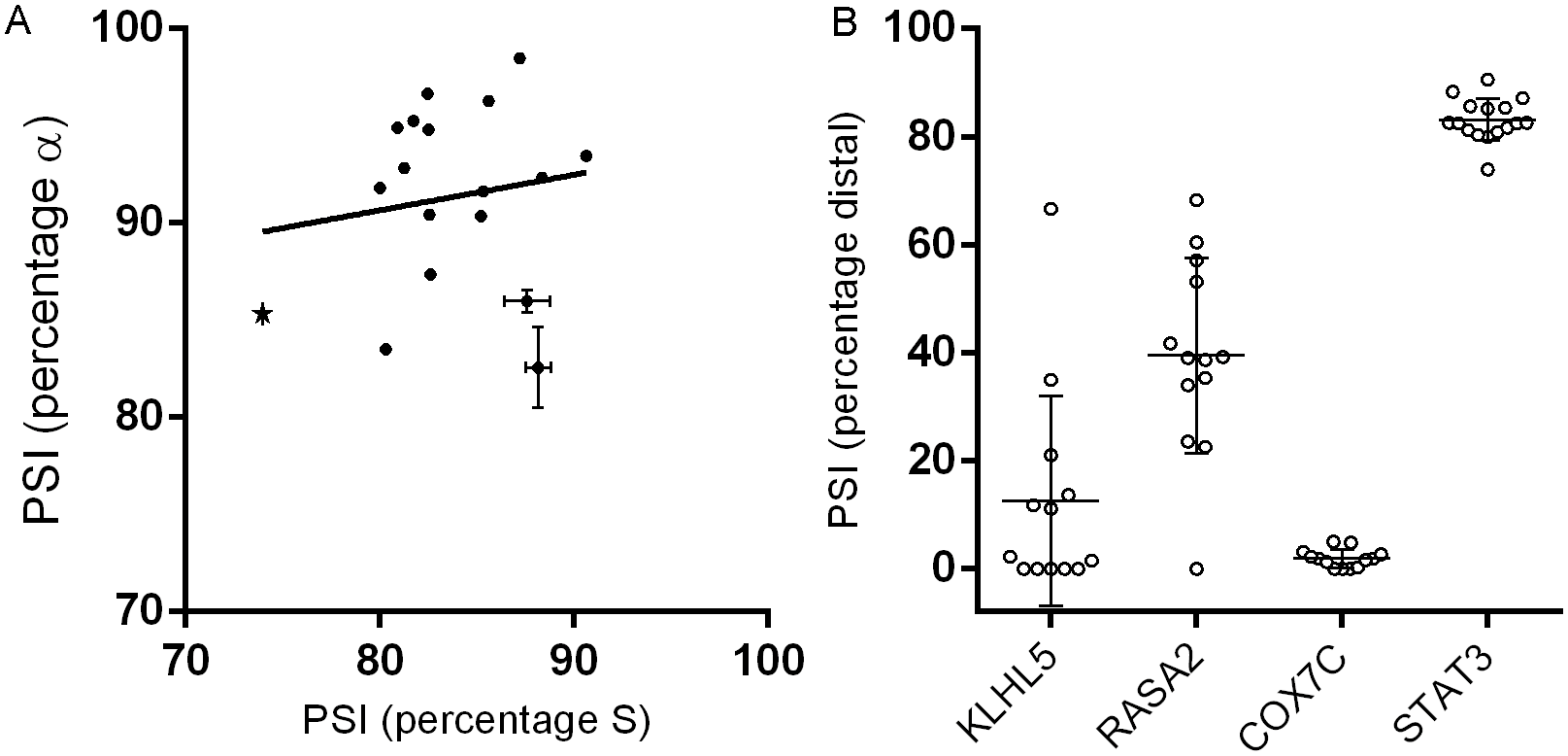
Analysis of “BodyMap” data. **(A)** *STAT3* PSI (percentage spliced in) values for at α/(α+β) vs S/(ΔS+S); with white blood cells denoted with a star. Our data based on qPCR are plotted with SEM. **(B)** Scatter Plot of PSI for 4 genes with GTAGTT donor splice sites. Data as mapped from Illumina BodyMap data across 16 tissues; within a minimum read count value of 15. Means and standard deviations are shown.

To provide further context of the analyses of eosinophils and DLBCL cells, we queried RNA-Seq data from the Illumina “BodyMap” 2.0 project [23] and retrieved percentage spliced-in (PSI) values of ΔS/S and α/β splicing across 16 analyzed tissues. S and α were more commonly spliced in all instances (Fig 7A). The variation in α/β (0.03–0.28) was greater than for ΔS/S (0.10–0.26). Regression analysis computed for proportions of ΔS and β in BodyMap tissue RNA-Seq revealed a possible trend that did not reach significance (r^2^ = 0.23, p = 0.06).

### ΔS/S ratios in tissues compared to other transcripts alternatively spliced at a GTAGTT donor splice site

An intron’s donor splice site is initially recognized by complementary sequences in U1 and U6 small nuclear RNA (for a review see [24]). Based on their canonical binding sites, U1 would prefer the ΔS sequence (CAG/gtagtt), while U6 binding would favor the S inclusion sequence (GTA/gttgtt) [24]. The nature of the overlapping binding sites means that splicing at tandem donor sites that includes or excludes 4 nucleotides is more common than splicing that includes or excludes a single codon and therefore maintains frame [25]. However, there are plenty of examples of so-called GYNGYN sites as found in *STAT3* [14]. Splice variant composition of three other genes with a GTAGTT tandem splice donor site was evaluated using Illumina’s BodyMap 2.0 RNA-Seq data (*KLHL5*: NM_001007075.2; *RASA2*: NM_006506.3 and *COX7C*: NM_001867.2). The splicing ratios of *KLHL5* and *RASA2* transcripts were highly variable from tissue to tissue and did not correlate with *STAT3* or each other (Fig 7B). The proximal site (equivalent to ΔS) was preferred for many but not all tissues for *RASA2* (also a Ser indel) and *KLHL5* (insertion of stop codon). In the case of *KLHL5*, nonsense-mediated decay [26] may have affected the spliceform composition. The alternate splicing ratio of *COX7C*, which is as consistent as *STAT3’s*, occurs in the 3' UTR and favors the proximal rather than the distal site. Thus, the splicing of *STAT3* stands out in the comparisons in two ways: preference for the distal (S) site and being constant from tissue to tissue.

## Discussion

The aims of this study were to determine the prevalence of the ΔS-β splice variant of human *STAT3*, which at present is not described in RefSeq or UniProt, and to develop assays for measuring *STAT3* splice variants. Through a process of optimization, we were able to create and characterize primer-specific qPCR assays for specific quantification of *STAT3* splice variants. Primers with high melting temperatures were required to distinguish the GC-rich ΔS/S site. The cycling program ensured specificity, but resulted in assays that were not as sensitive as typical qPCR and suited only for absolute quantification rather than comparisons to reference genes. Overall variant ratios were relatively constant across samples studied, although one ABC DLCBL cell line (RIVA) had a significantly higher proportion of S-β. The *STAT3* ΔS-β splice variant comprised the smallest proportion of the four variants, but was present at a higher level than predicted given the α/β and ΔS/S ratios, indicating the two splicing events are not independent of one another. The average percentages for ΔS transcripts in eosinophils (17%) and lymphoma cell lines (12%) differed from the 26% that we found for ΔS through inspection of publicly available RNA-Seq data generated from white blood cells (WBC) (Fig 7B) Because eosinophils account for <4% total WBC and DLBCL cells are atypical lymphocytes, the differing percentages are not necessarily conflicting. The high level of *STAT3* S-β in RIVA cells may be representative of what is found in normal lymphocytes. In addition, the high proportion of STAT3β in the promyelocytic cell line HL-60, a trait believed to influence differentiation in response to cytokines [27], may be representative of the amount in normal neutrophils.

The JAK/STAT signaling pathway initiated by IL5-family cytokines is important for eosinophil survival [28], and the IL3+TNFα combination enhances phosphorylation of STAT5A and transcription of cell cycle regulatory genes [29]. Our pan-*STAT3* qPCR assay demonstrated a 7.7-fold increase in *STAT3* mRNA levels after 6 hr of IL3+TNFα stimulation. The individual splice variants all increased but to different degrees. More detailed studies with more biological replicates would be needed to determine if the stimulated eosinophils have preferential splicing of the S-α variant, or increased turnover of certain variants. For example, because translation of *STAT3β* isoforms terminates in the penultimate coding exon, these transcripts are susceptible to nonsense-mediated decay [26].

Inspection of publically available tissue-specific RNA-Seq data indicate that *STAT3*’s ΔS/S ratio is more constant than its α/β ratio, and strikingly more constant than alternative splicing of GTAGTT tandem splice donor sites in *KLHL5* and *RASA2*. Although *COX7C* splicing was as consistent as *STAT3’s*, COX7C favored the proximal splice donor site. Direct sequencing has previously demonstrated [14] that *STAT3* ΔS splice variants are less abundant than S splice variants in mice, rats and cows as well as in humans; that is, the ratio of the two variants is conserved among species. Frogs, rather than the splice variation, have two *STAT3* genes with and without the codon for the Ser-701 equivalent (NM_001096733.1 and NM_001086108.1). These findings taken together strongly suggest that STAT3 function in all tissues depends on having an appropriate ratio of S and ΔS. However, because tissues are composed of diverse sets of cells, there may be cell types which lack ΔS. The existence of a “splico-stat” to maintain splice variant stoichiometry has been proposed in respect to tandem acceptor site usage and suggested to contribute to gene dosage balance that in turn influences cell phenotype [30].

Several observations suggest potential important functions of having a mix of STAT3 proteins that have or lack Ser-701. In the crystal structure of the activated STAT3β dimer in which the SH2 domain of one monomer engages phosphorylated Tyr-705 on the other monomer, Ser-701 is in the unstructured linkers that stretch between the SH2 domains and phosphorylated Tyr-705s [31]. Absence of Ser-701 would shorten the linker and may influence dimer formation. In addition, the close proximity of Ser-701 to Tyr-705 may influence site recognition by kinases or phosphatases. Finally, mass spectrometry of peptides isolated by affinity to immobilized metal [32,33] or Cell Signaling Technology’s stable of anti-phosphosite antibodies [34] identified peptides in which Ser-701 itself was phosphorylated in the presence and absence of Tyr-705 phosphorylation. In these studies, a peptide was identified with phospho-Tyr-704, indicating that Ser-701 is not required for the activating phosphorylation event. Although several tandem splice site bioinformatics analyses have been conducted; little has been done to address the functional consequences of triplet tandem sites (single codon indels) at the protein level. STAT3 is a good candidate for such investigations. Active STAT3 exists as a dimer. If one accounts for ΔS splice variants, and STAT3’s ability to heterodimerize with STAT1α and β [35]; there are 18 possible STAT3 or STAT3/STAT1 dimer combinations; potentially with differing activities. Furthermore, the nuclear localization kinetics of activating STAT3α and β differ [36]. The proximity of Ser-701 to Tyr-705 (and Ser-701 phosphorylation) could mean the ΔS/S splicing decision has consequences for STAT3 dynamics. When one adds the presence or absence of Ser-701 and phosphorylation at Ser-701 to the phosphorylations at Thr-713/714 and Ser-726/727, the permutations of modifications about Tyr-704/705 become numerous.

Regulation of alternate splicing is known to depend on *cis* and *trans* elements [37]. Global bioinformatics analyses have allowed development of predictive algorithms for exon skipping, mutually exclusive exons, and conventional (>18nt apart) alternate acceptor/donor splice sites. The splicing code is based on DNA sequences (*cis*) of the splice sites and intronic regions; and known cell-type-specific splicing factors (*trans*), and is fairly robust for predicting typical conventional alternate donor and acceptor splicing [37]. The regulation of 3nucleotide tandem splice sites is more conjectural. Burge *et al*. [38] and Hiller *et al*. [14] have presented data supporting the hypothesis that selection is not simply stochastic. If splicing were dependent on sequence and tissue-specific splice factors, one would expect that GYNGYNs with the same sequence as *STAT3*’s tandem donor splice site would have comparable splicing patterns in the same tissues, which is not the case. Other acceptor site sequence and intronic elements may influence splicing but it seems unlikely that tissue-specific splice factors or the spliced sequence itself are the only factors predisposing the gene to a particular splicing outcome based on the comparison of splice variant compositions of *RASA2*, *KLHL5*, *COX7C* and *STAT3* across tissues (Fig 7B).

Our data suggest that the α/β and ΔS/S splicing events are not independent, as a higher proportion of β splice variants lacked the serine codon than the proportion of α splice variants lacking it. Thus, splicing at *STAT3*’s tandem donor site at the 3' end of exon 21 resulting in inclusion/exclusion of the Ser-701 codon appeared to be weakly coupled to the splicing at the alternate acceptor sites with different reading frames in exon 23 that leads to either *STAT3α* or *β*. The gene’s ΔS/S splice site intronic region is 5057/5060 bp, while the intronic region of α/β is 280 or 330 bp. The shared acceptor site for ΔS/S is less than 100 bp from the α/β splice donor site. To our knowledge, no precedent exists for evaluating biases in alternate donor/acceptor site combinations in non-adjacent exons in mammals; although interdependence of non-adjacent whole-exon splicing has been observed for fibronectin [39] and the physiological consequences of *slo-1* gene splicing coordination have been studied in *C*. *elegans* [22]. Cooperative recruitment of splicing factors has been put forward as a potential mechanism [39]. However, it is possible that the bias in the ratio is not due to coordinated splicing but due to the differential stability of the ΔS-β, for instance because of RNA secondary structure or microRNA specificity. Additionally, Ensembl’s STAT3 annotation (ENSG00000168610) reveals that it is a Type II gene in terms of alternative polyadenylation sites: the final exon’s 3' untranslated region has multiple possible polyadenylation cleavage sites (for a review see [40]). The co-ordination in splicing may extend to biasing the choice of proximal or distal polyadenylation sites in the 3' UTR of transcripts, which would also influence stability.

## Materials and Methods

### Ethics Statement

Peripheral human blood eosinophils were purified by Percoll centrifugation and negative selection for neutrophils and monocytes as described previously [41]. The cells were received without identifying information in accord with a protocol approved (#2013–1570) by the University of Wisconsin-Madison Center for Health Sciences Institutional Review Board. Signed informed consent from the donor was obtained for the use of each sample in research. Eight established DLBCL cell lines from the American Type Culture Collection were used; six ABC DLBCL (SUDHL2, RIVA, HBL1, Oci-LY3, Oci-LY10 and TMD8) and two GCB DLBCL (SUDHL7 and Oci-LY1).

### Preparation of cDNA from eosinophils and DLBCL cell lines

Eosinophils were treated with IL3 or IL3 and TNFα as described [29]. Lymphoma cells were maintained in RPMI supplemented with 20% FBS at 37°C with 5% CO_2_. RNA was extracted using the RNeasy kit (Qiagen, Henlo, Germany). DLBCL and eosinophil cDNA was generated using the SuperScript III First-Strand Synthesis System for RT-PCR kit (Invitrogen, Madison, WI). For DLBCL, this entailed adding oligo(dT) primers and dNTPs to each RNA sample, incubating at 65°C for 5 minutes whereupon Invitrogen’s cDNA Synthesis Mix was added, and incubated at 50°C for 50 minutes. The reaction was terminated by incubating at 85°C for 5 minutes. Remaining RNA was removed by incubating with RNase H at 37°C for 20 minutes. The eosinophil cDNA was generated using random primers with the rest of the protocol being the same.

### Sequencing of *STAT3α* and *β* splice variants in eosinophils

Primer sequences based on published sequences for *STAT3α* and *β* were used to amplify a region of the 3ˈ-end of the transcript, spanning the ΔS/S splice site; so that all four variant sequences could be amplified from eosinophil cDNA. A common forward primer (spanning exon junction 18–19: 5ˈ- ATC CTG GGT ACC TGG AAC GAA GGG TAC ATC ATG GG -3ˈ) was used, with reverse primers specific to α (exon 23: 5ˈ- GTT CTC GCT AGC TCA CAT GGG GGA GGT AGC GC -3ˈ) and β (exon junction 21-22b: 5ˈ- GCA CCT GCT AGC TTA TTT CCA AAC TGC ATC AAT GAA TG -3ˈ). Products were sequenced. Longer PCR products were generated with a forward primer to a sequence in exon 19 and reverse primer to a sequence in exon 23 with 5ˈ extensions with restriction sites for *NheI* and *KpnI*. The PCR product was purified, digested with *NheI* and *KpnI*, and cloned into pET-Elmer (variant of pET-28a) [42], transformed into *E*. *coli* strain DH5α, and sequenced to identify clones for the four variants.

### Quantitative PCR

Since *STAT3*’s two alternate splice sites are in close proximity, exon-exon junction-spanning primers that specifically amplified each splice variant, referred to as S-α, S-β, ΔS-α and ΔS-β, were designed using Primer Select (DNAStar, Madison, WI) software. Primers were designed based on sequences NM_139276.2 (*STAT3* S-α), NM_213662.1 (*STAT3* S-β) and NM_003150.3 (*STAT3* ΔS-α) in NCBI. Various combinations were tested to identify primers that could be used to amplify the four variants. Additionally, a primer pair that recognized a region present in all *STAT3* splice variants (spanning exons 19 and 20), and a primer pair for a housekeeping gene β-glucuronidase (*GUSB*: NM_000181.3) were used (Integrated DNA Technologies, Coralville, IA, Table 1).

Relative quantification (ΔΔCt method [21]) was used to determine relative *STAT3* expression across eosinophil samples, as *GUSB* expression is known to remain constant [29]. A comparison of *STAT3* levels between cell lines, rather than within one treated cell type would require the assumption that a housekeeping gene’s transcripts comprise very similar percentages of total transcripts in all the cell lines. Thus, we did not evaluate overall *STAT3* in DLBCL cell lines.

Absolute quantification was used to evaluate splice variant composition within each sample, since the restricted primer sites make efficiency optimization (necessary for relative quantification) difficult [43]. Serial dilutions of the plasmids described above were used as templates for amplification with splice variant-specific primers (Table 1). These values were used to generate standard curves of *Ct* vs log(copy number) for absolute quantification.

Both absolute and relative quantifications were performed using an Applied Biosystems 7500 Real-Time PCR System in 96-well optical reaction plates. For each reaction 2 μL cDNA (1:1 diluted) was added to a 23 μL reaction mixture containing 12.5 μL SYBR Green Master Mix (Life Technologies, Madison, WI) and 533 nM each of the forward and reverse primers. A threshold value of 0.200 was used for all experiments, with automatic baseline.

An optimized qPCR program balancing specificity and sensitivity was used for splice variant (absolute) quantification:

$$50{}^{\circ}C\;(2\;minutes)\;\to \;95{}^{\circ}C\;(5\;min)\;\to \;40\times \left\{95{}^{\circ}C\;(15\;sec)\;\to \;66{}^{\circ}C\;(30\;sec)\;\to \;72{}^{\circ}C\;(1\;min)\right\}\to \;95{}^{\circ}C\;(15\;sec)\;\to \;60{}^{\circ}C\;(1\;min)\;\to \;95{}^{\circ}C\;(15\;sec)$$

The post-amplification dissociation phase data were used to ensure no genomic DNA was being amplified. Conventional PCR was performed with the same program (without the dissociation phase after amplification) during optimization, using GoTaq polymerase (Promega, Madison, WI), with 1.5 mM MgCl_2_.

For (relative) qPCR with pan-*STAT3* and *GUSB* less stringent conditions were required to achieve specificity. Efficiencies were within the 98–102% range; which meant data were amenable to relative quantification. Primer concentrations were 400 nM, but other components were as above.

$$50{}^{\circ}C\;\left(2\;minutes\right)\to \;95{}^{\circ}C\;\left(5\;min\right)\to \;\left|95{}^{\circ}C\;\left(15\;sec\right)\to \;60{}^{\circ}C\;\left(1\;min\right)\right|\to \;95{}^{\circ}C\;\left(15\;sec\right)\to \;60{}^{\circ}C\;\left(1\;min\right)\to \;95{}^{\circ}C\;\left(15\;sec\right)40x$$

Negative controls (as used in conventional PCR) were included to verify that the primers were amplifying specifically. Too’s formula [44] for qPCR specificity (σ) was used for each splice variant:

$$\sigma \;=\;{\left(10-1/slope\;of\;standard\;curve\right)}^{{\Delta Ct}^{Too}}$$

(in this equation *ΔCt* ^*Too*^ = *Ct* ^*target*^-*Ct* ^*non*-*target*^)

Since “ΔS” vs “S” needed the most stringent conditions to achieve specificity in conventional PCR; Δ*Ct* was measured with “non-target” template S-α being amplified with a “target” ΔS-α specific primer combination; and vice versa. The β splice variants were tested similarly. All assays had greater than a 10^4^-fold specificity for their splice variant, the same range recorded by Too [44].

Sensitivity was examined based on lowest copy number detectable. The assay reliably detected *STAT3* splice variants when plasmid copy numbers exceeded 10^2^ per well; with an interassay CV of <4%. At lower concentrations, the *Ct* value was less consistent. Only *Ct* values smaller than 38 were considered reliable. In comparable investigations with less similar splice variants, 10^1^ copies per well were detectable [45].

To assess efficiency, slopes of *Ct* vs log(copy number) for the four splice variant standards (plasmid calibrators) were plotted. These ranged from -3.78 to -3.47. The y-intercept values ranged from 39.5 to 42.2 between splice variants. Although -3.78 is not ideal (efficiency 82%), it is acceptable for the purpose of absolute quantification [43]. The efficiency of amplification was determined in sample cDNA as well, to verify that absolute quantification was a valid means of comparing splice variant levels. Standard curves of serially-diluted samples were generated under otherwise identical conditions to calibrator plasmids’ curves. Two of the eosinophil cDNA samples were tested, and two of the DLBCL samples.

In accordance with the MIQE guidelines [46] the repeatability (intra-assay) and reproducibility (interassay) of each measurement were recorded. Briefly, samples were analyzed in duplicate per plate for a particular splice variant/gene, and were analyzed on at least two occasions. For splice variant studies, these were determined as coefficient of variation (CV) of the total amount of splice variant. All CVs within and between assays for DLCBL cDNA were <15.3. If *Ct* values of duplicates differed by more than 0.5, the reaction was repeated.

The standard deviations (SD) between intra-assay *Ct* values were calculated as a measure of repeatability in relative quantification (pan-*STAT3* and *GUSB*) assays. The *Ct* SDs between duplicate samples did not exceed 0.2. Furthermore, ΔΔ*Ct* values measured in two separate assays were within 1 SD of each other.

### Statistics

Differences in STAT3 mRNA and splice ratio were analyzed for significance using a two-tailed paired Student’s *t*-test. A 90% confidence interval for pan-*STAT3* vs. sum of splice variants was calculated to test equivalence. All statistics were computed using Prism GraphPad software.

### Probing publicly-available RNA-Seq data

We mapped intron-spanning reads from Illumina BodyMap 2.0 project [23] (NCBI GEO accession: GSE30611) to human genome build hg19 using Integrative Genome Viewer [47]. From these data, percentage spliced in (PSI) values could be determined for the α/β and ΔS/S splice sites of *STAT3*; and for the tandem donor splice sites of *KLHL5*, *COX7C* and *RASA2*.

## Supporting Information

S1 Data The raw Ct values and workflow used to analyze both absolute and relative qPCR data are included as a supplementary Excel document. (XLSX)
